# The Role of NcRNAs to Regulate Immune Checkpoints in Cancer

**DOI:** 10.3389/fimmu.2022.853480

**Published:** 2022-04-06

**Authors:** Yicun Jiang, Leilei Zhao, Yiwen Wu, Sijun Deng, Pu Cao, Xiaoyong Lei, Xiaoyan Yang

**Affiliations:** ^1^ School of Pharmacy, Hengyang Medical College, University of South China, Hengyang, China; ^2^ Hunan Provincial Key Laboratory of Tumor Microenvironment Responsive Drug Research, University of South China, Hengyang, China

**Keywords:** miRNA, circRNA, lncRNA, cancer, immune checkpoints

## Abstract

At present, the incidence of cancer is becoming more and more common, but its treatment has always been a problem. Although a small number of cancers can be treated, the recurrence rates are generally high and cannot be completely cured. At present, conventional cancer therapies mainly include chemotherapy and radiotherapy, which are the first-line therapies for most cancer patients, but there are palliatives. Approaches to cancer treatment are not as fast as cancer development. The current cancer treatments have not been effective in stopping the development of cancer, and cancer treatment needs to be imported into new strategies. Non-coding RNAs (ncRNAs) is a hot research topic at present. NcRNAs, which include microRNAs (miRNAs), circular RNAs (circRNAs), and long non-coding RNAs (lncRNAs), participate in all aspects of cancer biology. They are involved in the progression of tumors into a new form, including B-cell lymphoma, glioma, or the parenchymal tumors such as gastric cancer and colon cancer, among others. NcRNAs target various immune checkpoints to affect tumor proliferation, differentiation, and development. This might represent a new strategy for cancer treatment.

## Introduction

The current treatments for tumors are not suitable for the increasingly evolving forms of cancer. Although new therapies that target proteins such as antibodies against programmed cell death 1 (PD-1), programmed death-ligand 1 (PD-L1), and cytotoxic T-lymphocyte-associated protein 4 (CTLA-4) have ushered in a new era in cancer pharmacotherapy and drug development, some oncogene-encoded protein targets are intractable or insufficient for achieving remission. In addition, cancer cells can develop drug resistance (1). As a result, successful cancer treatment necessitates several types of targets implicated in carcinogenic pathways.

Non-coding RNAs (ncRNAs) are transcripts that do not encode proteins, except the annotated messenger RNAs (mRNAs) (2). NcRNAs play a role in various aspects of cancer biology, including cell death, proliferation, and treatment resistance (3). There are three different types of ncRNAs, namely, microRNAs (miRNAs), circular RNAs (circRNAs), and long non-coding RNAs (lncRNAs). MiRNAs are short non-coding regulatory RNAs that regulate protein expression by binding to the 3′-untranslated region (3′-UTR) of mRNA and limiting mRNA translation while also altering transcription (4). Some concrete miRNA systems are eminently related to the initiation and progression of cancer, as well as drug resistance (1, 5). CircRNAs are covalently closed-loop ncRNAs without the 5′ and 3′ ends, and they are created by the back-splicing of pre-mRNAs (6). Additionally, they measure a category of ncRNAs or protein-coding RNAs derived from one coding DNA or multiple exons and are located within the living substance (7, 8). CircRNAs act as miRNA sponges to modulate the organic phenomenon and have an effect on the biological processes of cancers (9). Previous studies showed that circRNAs can take part in many physiological and pathological courses, including tumorigenesis and the progression of cancer (10–12). LncRNAs are RNAs with more than 200 nucleic acids and have the specialty of evolution and expression of tissue. They are abnormal in a lot of carcinomas and participate in post-transcriptional shearing, editing, and in the transportation, translation, and degradation of post-transcription (13, 14).

Immune checkpoints can measure the level of immune activation. They are a class of molecules that are expressed on immune cells. The growth and the progression of cancer are related to immune suppression (15). Over the past decade, the inhibition of immune checkpoint inhibitors has increasingly propelled the event of cancer medical specialty (16–18).

**Figure 1:**
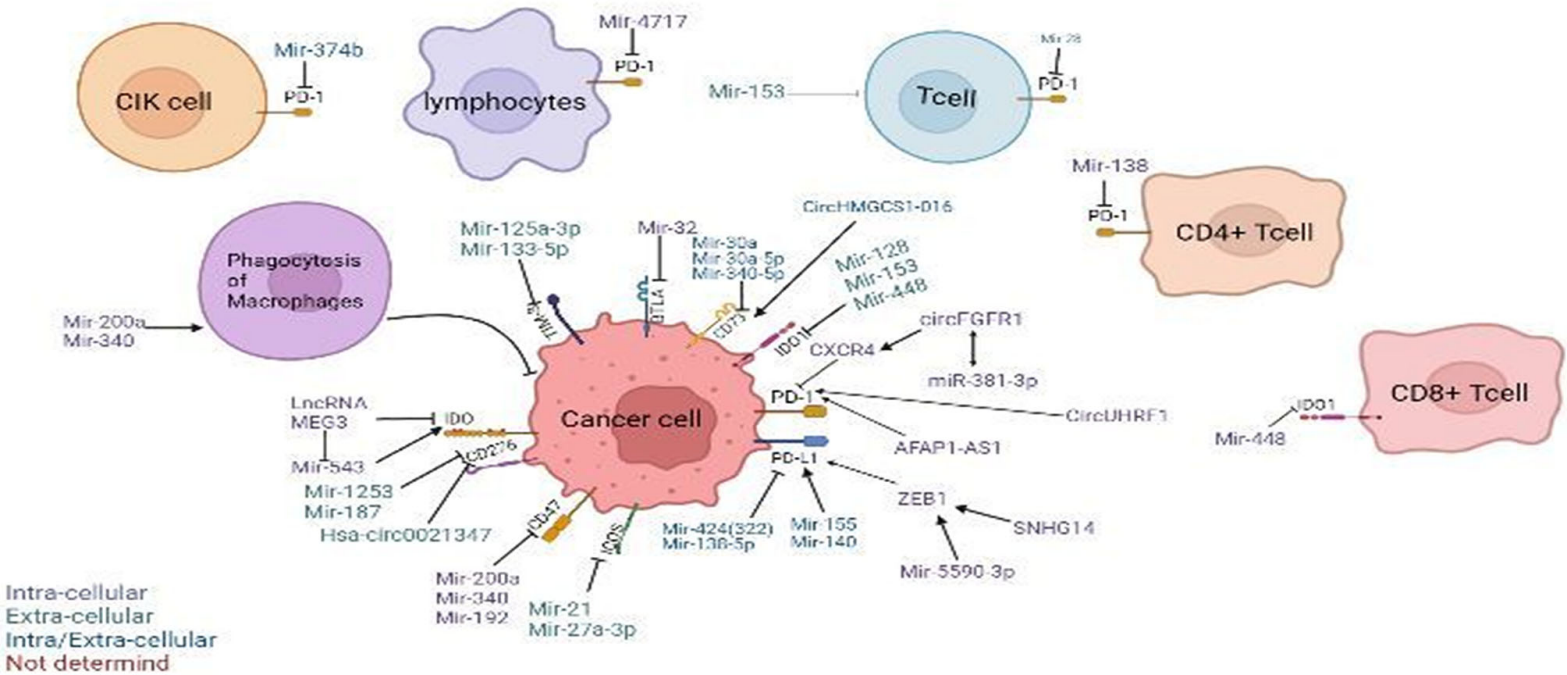
Regulation of non-coding RNAs (ncRNAs) in the tumor microenvironment. Modulatory effects of ncRNAs on cytokine-induced killer (CIK) cells and phagocytosis of macrophages. ncRNAs regulate different immune checkpoints, including PD-1, PD-L1, CD47, CD73, IDO, IDO1, TIM-3, BTLA, ICOS, and B7-H3(CD276), in cancer cells, T cells, T lymphocytes, CD4^+^ T cells, and CD8^+^ T cells. *Arrow* denotes stimulatory regulation, while *T* denotes inhibitory regulation. The *colored legend on the bottom left panel* represents the sites of action of the depicted immunomodulatory ncRNAs.

There are reports showing that blocking the expression of immune checkpoints can have an impact on the development of cancer (15), but the current research works have not been clear on its specific mechanism. This paper mainly expounds on the role of ncRNAs in immune checkpoints the specific mechanisms of microRNAs target immune checkpoints in cancer can be showed in **Table 1**, the mechanisms of circular RNAs target it in cancer can be showed in **Table 2**, and that of Long non-coding RNAs target in cancer can be showed in **Table 3**, thus making explicit the incidence and development of human cancers in order to provide new perspectives for their treatment. The specific mechanisms of the three types of ncRNAs in different immune checkpoints are shown in **Figure 1**.

**Table 1:** MicroRNAS (miRNAs) target immune checkpoints in cancer.

MiRNA	Target	Direction of misregulation	Host	Reference
MiR-155	PD-1/PD-L1	Up	B-cell lymphoma	(19)
MiR-140	PD-L1	Up	GC	(20)
MiR-424 (322)	PD-L1	Down	OC	(21)
MiR-138	PD-1	Down	Glioblastoma	(22)
MiR-374b	PD-1	Down	Liver cancer	(23)
MiR-4717	PD-1	Down	HCC	(24)
MiR-28	PD-1	Down	Melanoma	(25)
MiR-340	CD47	Down	PADC	(26)
MiR-200a	CD47	Down	NPC	(27)
MiR-192	CD47	Down	Medulloblastoma	(28)
MiR-218	IDO1	Down	Cervical cancer	(29)
MiR-153	IDO1	Down	Colon cancer	(30)
MiR-448	IDO1	Down	Colon cancer	(31)
MiR-32	BTLA	Down	OC	(32)
MiR-30a	CD73	Down	CRC	(33)
MiR-30a-5p	CD73	Down	NSCLC	(34)
MiR-340-5p	CD73	Down	DLBCL	(35)
MiR-133-5p	TIM	Down	AML	(36)
MiR-125a-3p	TIM	Down	AML	(37)
MiR-21	ICOS	Down	B-cell lymphoma	(38)
MiR-27a-3p	ICOS	Down	LUAD	(39)
MiR-1253	CD276	Down	Medulloblastoma	(40)
MiR-187	CD276	Down	CRC	(41)

GC, gastric cancer; OC, ovarian cancer; HCC, hepatocellular carcinoma; PADC, pancreatic ductal adenocarcinoma; NPC, nasopharyngeal cancer; CRC, colorectal cancer; NSCLC, non-small cell lung cancer; DLBCL, diffuse large B-cell lymphoma; AML, acute myeloid leukemia; LUAD, lung adenocarcinoma.

**Table 2:** Circular RNAs (circRNAs) target immune checkpoints in cancer.

CircRNA	Target	Direction of misregulation	Host	Reference
CircFGFR1	PD-1	Down	NSCLC	(42)
CircUHRF1	PD-1	Up	HCC	(43)
CircRERE	CD47	Up	MM	(6)
CircHMGCS1-016	CD73	Up	ICC	(44)
Hsa-circ0021347	CD276	Down	OS	(45)

NSCLC, non-small cell lung cancer; HCC, hepatocellular carcinoma; MM, myeloma; ICC, intrahepatic cholangiocarcinoma; OS, osteosarcoma.

**Table 3:** Long non-coding RNAs (lncRNAs) target immune checkpoints in cancer.

LncRNA	Target	Direction of misregulation	Host	Reference
LncRNA *MEG3*	IDO	Down	NSCLC	(46)
LncRNA *AFAP1-AS1*	PD-1	Up	NPC	(47)
*SNHG14*	PD-1/PD-L1	Up	DLBCL	(48)

NSCLC, non-small cell lung cancer; NPC, nasopharyngeal cancer; DLBCL, diffuse large B-cell lymphoma.

## The Impact of NcRNAs Targeting PD-1/PD-L1 on Cancer

PD-1 and PD-L1 intercede the immunological disorder effects of tumors through encouraging T-cell apoptosis and the induction of regulatory T cells (Tregs) (49). The anticancer effects of antibody treatments targeting PD-L1 and PD-1 have been demonstrated, which are similar to those of CTLA-4 by reversing the immunosuppressive effects of PD-1 (50).

### MiRNAs Target PD-1/PD-L1

MiR-155 is closely related to the progression of B-cell lymphoma. In the tumor microenvironment, it enhances the interaction between B-lymphoma cells and CD8^+^ T cells, which may be directly targeted by the inhibition of PD-1/PD-L1 (19). In the spleen and bone marrow, miR-155 transgenic mice exhibited pre-B-cell proliferation and also showed malignant B-cell transformation (51). In B-lymphoma cells, miR-155 increased the expression of PD-L1 through directly combining with the 3′-UTR, aggravating CD8^+^ T-cell necrobiosis, and preserving neoplasm immunity in a particularly PD-1/PD-L1-dependent manner (19). The AKT and ERK pathway is the key regulator of PD-1-mediated CD8^+^ T-cell function (52). In DB cells, ectopic expression of miR-155 caused an increased sensitivity to anti-PD-L1 and anti-PD-1 antibodies (19). In nude mice, the downregulation of miR-155 promoted the apoptosis of B-lymphoma cells and delayed xenograft tumor formation (53). In summary, miR-155 may be a potential target for anti-PD-L1 protein treatment of malignant B-cell neoplastic diseases.

MiR-140 played a suppressive role in the growth of gastric cancer *in vitro* and *in vivo* (54, 55). It also strengthened antitumor immunity in gastric cancer (20). PD-L1 was directly targeted by miR-140 in gastric cancer cells (20). Previous studies showed that cell-intrinsic PD-L1/PD-1 signaling promoted tumor development by activating downstream mammalian target of rapamycin (mTOR) signaling (56). Previous research also showed that miR-140 played a suppressive role in mTOR signaling in gastric cancer (57). Moreover, miR-140 can reduce the expression of PD-L1 (20). Signaling inhibition of PD-L1/PD-1 was able to stop immune suppression and promote antitumor response (58). By targeting PD-L1 *in vitro* and *in vivo*, the upregulation of miR-140 can inhibit gastric cancer growth; it is also related to an enhanced antitumor immunity (20). Consequently, miR-140 may represent a new therapeutic target for the treatment of gastric cancer through targeting PD-L1.

MiR-424(322) was conversely correlated with the expressions of *PD-L1*, *PD-1*, *CD80*, and *CTLA-4* in a clinical gene expression array dataset (21). Through direct binding to the 3′-UTR of these genes, the overexpression of miR-424(322) inhibited the expressions of *PD-L1* and *CD80* (21). It has been proven that the PD-L1/PD-1 immune checkpoint pathway can inhibit the T-cell antitumor immune response (59), and PD-L1 preferentially modulates the secretion of regulator cytokines in T cells (60). Restoration of the expression of miR-424(322) in female internal reproductive organ cancer chemoresistant cells caused a decrease in the expression of PD-L1 (21). Blockade of PD-L1 restored immune suppression and caused the expansion of tumor-infiltrating lymphocytes (TILs) (61). Restoration of the expression of miR-424(322) increased the sensitivity of cancer cells to drug treatment and activated T cells through blocking PD-L1 in *in vivo* and *in vitro* models (21). All the above indicate that miR-424(322) can target PD-1 and be involved in the treatment of ovarian cancer.

With PD-1 and CTLA-4, various miRNA-targeting algorithms have indicated the possible interactions of miR-138 (22). There is an explicit variety of miRNAs within the brain, and miR-138 is said to regulate nerve fiber spine formation in rat hippocampal neurons (62). The accumulation of relevant information cumulatively revealed that, *in vivo*, miR-138 preferentially modulates the immune system, especially interacting with PD-1 and CTLA-4 to inhibit tumor-infiltrating Tregs and later alleviating the damage by immunological disorder cells in the tumor microenvironment (22). In glioma, miR-124 enhanced the T-cell-mediated immune clearance by inhibiting STAT3 signaling (63). Therefore, the hypothesis that miR-138 could regulate Tregs has been proven and that the administration of miR-138 *in vivo* could exert potent antitumor immune effects through interacting with CTLA-4 and PD-1 (22). This could be a promising idea for glioma treatment.

The transforming gene *PD-1* is related to the suppression of proliferation and protein production of T cells in the progression of liver disease (23). The downregulation of PD-1 was revealed to be able to reverse immune dysfunction (64–66). In T cells, PD-1 acts as a regulator inhibiting the proliferation and cytokine production (67). Relevant incontestable knowledge revealed that metallic element one could be a real target of miR-374b (23). In B cells, miR-374b is associated with the acceleration of cell proliferation and, therefore, the production of aberrant glycosylated antibody (immunoglobulin, Ig) A1 (68). One study significantly showed that miR-374b mimics played a suppressive role in the mRNA and macromolecule expression of PD-1 in cytokine-induced killer (CIK) cells and that it also has influence on the tumor-targeting capability of CIK cells (23). The interaction between PD-1 and miR-374b may offer some new ideas on the design of new therapeutic modalities for liver cancer treatment.

PD-1 plays a prominent role in the regulation of T-lymphocyte function and could be a main issue of cell death sensitivity (69, 70). More and more research studies have demonstrated that the PD-1 pathway and its ligands to be basically concerned in virus infection, as well as HBV infection (24). PD-L1 is induced by viral infection, interferon (IFN)-α, and IFN-γ of hepatocytes and mediates T-cell apoptosis (71). The SNP rs10204525 has been connected with chronic HBV infection (25, 72, 73), and the *PD-1* gene is located in the 3′-UTR (24). Additionally, miR-4717 allele-specifically regulates the expression of PD-1 through a totally different interaction with its polymorphic target within its 3′-UTR. This also means that miR-4717 plays a role in the susceptibility to chronic HBV infection (24). The relationship between PD-1 and miR-4717 may provide new ideas for HBV treatment.

Experimental data from melanoma-bearing mice suggested that miR-28 has the potential to regulate the *PD-1*, *TIM3*, and *BTLA* gene in T cells (74). In the cancer environment, high levels of overexpressed inhibitory receptors are among the main features of T-cell exhaustion (75). Dual-luciferase assay of the PD-1 3′-UTR showed that miR-28 directly silenced PD-1 *via* binding to its 3′-UTR (74). Moreover, PD-1 has been recognized because of the primary biomarker for T cells that are used up (76). It can be inferred that miR-28 depletes T cells by silencing PD-1 and that it has an antitumor effect.

### CircRNAs Target PD-1

Fibroblast protein receptor 1 (FGFR1) is a type of oncogene that may promote the progression and tumorigenesis of non-small cell lung cancer (NSCLC) cells using totally different strategies (77). CircFGFR1 can also promote resistance of NSCLC cells to anti-PD-1 agents. In xenograft mice, overexpression of circFGFR1 could significantly reduce PD-1 in Lewis lung carcinoma (LLC) cells. The tumor growth in LLC-circFGFR1 cell recipient xenograft mice showed obvious phenotypic resistance to anti-PD-1 therapy compared with that of the mock cell group, and xenograft mice had a shorter survival time (42). Previous research studies showed that forced circFGFR3 expression competitively binds to miR-22-3p to promote NSCLC cell invasion and proliferation and facilitate galectin-1 activity (78). CXCR4 could be a possible intermediator that guides cytotoxic T-lymphocyte exclusion and participates in the resistance to anti-PD-1 cancer therapeutic methods (79, 80). The expression of circFGFR1/CXCR4 and the frequency of CD8^+^ T cells showed a negative correlation in NSCLC tissues (42). Furthermore, NSCLC cells are sensitive to anti-PD-1 immunology therapy after knockout of CXCR4 (42). Therefore, inhibiting the circFGFR1/CXCR4 pathways in NSCLC cells may provide a novel opportunity to inhibit resistance to anti-PD-1 immunotherapy in the treatment of NCSLC.

Researchers have found that forced UHRF1 expression can promote tumorigenesis and the progression of hepatocellular carcinoma (HCC) (81). It was indicated that circRNA from the expression of UHRF1 can be upregulated and could possibly promote the progression of HCC (43). In HCC patients, overexpression of PD-1 in natural killer (NK) cells can promote the functional dysregulation of activated NK cells (82, 83). Exosomal circUHRF1 secreted by HCC cells induced NK-cell exhaustion and mediated the resistance to anti-PD-1 therapy (43). In another study, it was shown that, compared to HCC patients susceptible to anti-PD-1 therapy, the number of NKG2D-positive cells in HCC patients resistant to anti-PD-1 therapy was significantly reduced. In addition, the results of a xenograft model by subcutaneous implantation showed that circUHRF1 knockdown cells led to sensitivity to anti-PD-1 treatment and to an increase in the overall survival rate (43). All these show that forced circUHRF1 expression might prevent the response of HCC to anti-PD-1 therapy.

### LncRNAs Target PD-1/PD-L1

By utilizing the Gene Expression Omnibus (GEO) dataset, a previous study found that the key molecular marker of growth immune evasion PD-1 was completely related to the expression of *AFAP1-AS1* in nasopharyngeal cancer (NPC) and that they are co-expressed in infiltrating lymphocytes in NPC tissues (47). In recent studies, PD-L1 and PD-1 have shown high expression levels in NPC (84–86). In order to regulate the expression of PD-1, *AFAP1-AS1* functions as a competing endogenous RNA (ceRNA) (87–89). The expression level of the lncRNA *AFAP1-AS1* was high in NPC, and it also promoted the violation and metastasis of cancer (47). All the above show that AFAP1-AS1 and PD-1 are great therapeutic targets for suppressing the metastasis of tumor and activating antitumor immunity.

Zinc finger E-box-binding homeobox gene 1 (*ZEB1*) is a transcriptional factor (TF) demonstrated to be associated with gene control in numerous varieties of cancer cells regarding invasion, migration, epithelial–mesenchymal translation (EMT), and proliferation (90–92). Small nucleolar RNA host gene 14 (*SNHG14*) elicits oncogenic functions through modulating proliferation, migration, and invasion to confer chemoresistance in different malignancies (48). Previous research works showed that ZEB1 can improve immune evasion by upregulating the expression of PD-L1 in cancer cells (93, 94). The ribonucleic acid expression of ZEB1, and therefore the macromolecule expressions of ZEB1 and PD-L1, in diffuse large B-cell lymphoma (DLBCL) cells was reduced under SNHG14 silencing (48). Through PD-1/PD-L1, SNHG14/ZEB1 evoked the interaction of DLBCL cells with CD8^+^ T cells and triggered apoptosis (48). This means that the interaction of SNHG14/ZEB1 and PD-1/PD-L1 might be a promising target in DLBCL treatment.

## The Impact of NcRNAs Targeting CD47 on Cancer

CD47 is a transmembrane supermolecule that functions as a ligand for signal regulatory protein alpha (SIRPα) and is additionally widely expressed on all traditional cells and overexpressed on growth cells (95).

### MiRNAs Target CD47

MiR-340 has been demonstrated to regulate the expressions of genes related to tumor progression and is involved in tumor suppression (96, 97). A study showed that, at both the mRNA and protein levels in Panc02 cells, the overexpression of miR-340 by mimic transfection significantly decreased CD47, especially in pancreatic ductal carcinoma (PDAC) (26). CD47 is a direct target of miR-340 and is downregulated by miR-340 through a binding site in its 3′-UTR (26). The functions of anti-CD47 include the inhibition and causation of efficient “self” signal uptake of cancer cells (98). Phagocytosis by macrophages was greatly enhanced in miR-340-overexpressed tumor-bearing mice, and the overexpression of miR-340 further increased with CD47 blockade (26). Therefore, miR-340 targeting CD47 may be a good option for PDAC treatment.

MiR-200a is one of the important factors in the miR-200 unit, which has five other members: miR-200a/b/c, miR-141, and miR-429 (99–101). MiR-200a is downregulated while CD47 is upregulated in NPC. The expression of miR-200a was negatively correlated with the levels of CD47 using Spearman’s correlation analysis (27). MiR-200a inhibited the expression of CD47, similar to small interfering RNA (siRNA) CD47, and restrained the macromolecular translation and reduced the expression of CD47 (27). CD47–SIRPα blockade can enhance the phagocytic activity of phagocytes to eliminate tumor cells (102). Long-standing research has provided proof that, with the activation of CD47 signaling, cancer cells will impair the immune system and evade the antitumor function of macrophages (103, 104). Studies also showed that miR-200a suppressed the proliferation, colony formation, migration, and invasion of NPC cells by downregulating CD47 (27). All these can provide potential novel therapy for NPC with the use of miR-200a/CD47.

MiRs that play suppressive roles in tumors are downregulated in cancer, contrary to the oncogenic ones (105). Compared to the normal cerebellum, the expression level of miR-192 in medulloblastoma cells is lower, and studies indicated that miR-192 suppresses the expressions of ITGAV, ITGB1, ITGB3, and CD47 in medulloblastoma (28). Previous studies showed that miR-192 is a metastasis suppressor gene (106, 107). ITGAVB3 has shown positive correlation with the adhesion ability of cells, which means that it has increased metastatic potential (108, 109). In addition, research works showed that the suppression of miR-192-mediated CD47 restrained leptomeningeal dissemination of medulloblastoma with molecular crosstalk with ITGAVB3 (28). All of these provide new perspectives in medulloblastoma treatment.

### CircRNAs Target CD47

CircRNA arginine–glutamic acid dipeptide repeat (circRERE) is a natural sponge of miR-152-3p, and the miR-152-3p inhibitor attenuated the suppressive effect of circRERE on bortezomib (BTZ) resistance (6). CD47 is associated with the degree of immune deactivation, and the therapeutic strategy concerning anti-CD47 has been employed to kill myeloma (MM) cells (110, 111). In accordance with its sponge effects on miR-152-3p, circRERE modulates the resistance of BTZ in MM cells through suppressing miR-152-3p (6). Overexpression of miR-152-3p resulted in the repression of the IC_50_ of BTZ and its cell proliferative ability, but accelerated cell apoptosis, which was then abolished by promotion of the expression of CD47. Protein detection by Western blot showed that, in 8226R5 and MM1, the upregulation of CD47 counteracted the miR-152-3p mimic-induced pro-apoptotic and anti-proliferative effects (6). BTZ is a first-line chemotherapeutic agent for MM, but treatment frequently fails due to the resistance to BTZ (112). Moreover, CD47 is a downstream target of miR-152-3p, and miR-152-2p inhibits BTZ resistance by reducing the levels of CD47 in MM cells (6). Downregulation of circRERE may be a new therapeutic target by inducing BTZ resistance in MM treatment.

## The Impact of NcRNAs Targeting IDO1 on Cancer

Indoleamine 2,3-dioxygenase 1 (IDO1), IDO2, and tryptophan 2,3-dioxygenase (TDO) are the initial and rate-limiting enzymes of the tryptophan metabolism pathway (113, 114). These enzymes convert essential amino acids into kynurenine and 3-hydroxyanthranilic acid. Essential amino acids are vital aminoalkanoic acids for the survival, proliferation, and activation of nerve fiber cells and lymphatic cells (30).

### MiRNAs Target IDO1

A study has shown miR-218 directly targeting the 3′-UTR of IDO1, and in cervical cancer tissues and cells, miR-218 was downregulated and negatively related to IDO1 (29). T cells in the G1 phase have high sensitivity to tryptophan deficiency, and IDO1 can lead to a significant lack of tryptophan. IDO overexpression may suppress the proliferation of T cells (115, 116). The overexpression of miR-218 considerably suppressed the expression of IDO1, inhibited cell viability, and promoted the caspase-mediated cell death of cervical cancer cells through the transfection of miR-218 mimics into HeLa cervical cancer cells (29). All the above can provide novel measures for the treatment of cervical cancer and some other tumors.

Cancer cells express higher levels of immunosuppressive molecules after interferon gamma (IFN-γ) induction and then escape immune elimination (117). It is believed that the upregulation of IDO1 by IFN-γ in neoplasm cells can cause immunological disorder in the tumor microenvironment, thereby restricting cytotoxic T cells (118). MiR-153 plays a suppressive role in tumors (119), which can directly target and downregulate the expression of IDO1 in colon cancer cells (30). It also limits the upregulation of IFN-γ on IDO1 and displays an antitumor effect.

IFN-γ strongly induced IDO1 in tumor cells (117). MiR-448 targeted IDO1, as shown by the results of dual-luciferase reporter assay and WB assay (31). MiR-448 inhibited the protein expression of IDO1 in human colon cancer (31), while CD8^+^ T cells greatly induced IDO1 (117). In addition, CD8^+^ T cells circulate in the blood and play a cytotoxic role (120). Previous studies showed that miR-448 blocked the expression of IDO1 to suppress the apoptosis of CD8^+^ T cells (31). This might provide insights for the treatment of colon cancer.

### LncRNAs Target IDO

LncRNAs are molecular markers of tumor diagnosis and prognosis (46, 121). A study showed that the lncRNA *MEG3* had a low expression and functioned as a tumor suppresser in NSCLC (32). In NSCLC cells overexpressing *MEG3*, the levels of IDO1, IDO2, TDO, and the autophagy-related Beclin-1 and LC3-II proteins were apparently reduced, whereas that of the autophagy-related LC3-I supermolecule was obviously enhanced (122). *MEG3* regulates the miR-543/IDO signaling pathway to influence the autophagy of lung carcinoma cells (32). Regulation of the miR-543/IDO signaling pathway by *MEG3* can be a treatment option for NSCLC.

## The impact of MiRNA Targeting BTLA on Cancer

The expression of MiR-32 is markedly lower in ovarian cancer tissues than that in adjacent normal tissues; miR-32 also directly targets the 3′-UTR of BTLA (123). BTLA is a new member of the CD28 family (34). Previous research showed that miR-32 can suppress the malignant behavior of HeLa cells (124). Moreover, the high expression level of BTLA could significantly play an anticancer role in miR-32, while the overexpression of miR-32 could greatly inhibit the mRNA and protein expressions of BTLA (123). A negative feedback loop was formed between miR-32 and BTLA, regulating the malignant behavior of ovarian cancer cells (123). The low expression levels of MiR-32 and its target on BTLA in ovarian cancer cells can help inspire treatment options for ovarian cancer.

## The Impact of NcRNAs Targeting CD73 on Cancer

Ecto5′-nucleotidase (CD73), a component of the purinergic signaling pathway, is a 70-kDa glycosylphosphatidylinositol-anchored cell surface supermolecule encoded by the *NT5E* cistron that is crucial in adenosinergic signaling (125).

### MiRNAs Target CD73

It was shown that miRNAs are closely related to the pathogenesis of cancers, such as cancer proliferation (33). CD73 can play an important part in cancer development (126). CD73 is a direct target gene of miR-30a in colorectal cancer (CRC) cells, while miR-30a showed a negative effect in the regulation of the expression levels of CD73 mRNA and protein (127). The effect of miR-30a overexpression can be reversed by the reexpression of CD73, and miR-30a inhibits the cell proliferation and tumor growth of body part cancer through targeting CD73 (127). The interaction between CD73 and miR-30a may be a promising approach for CRC treatment.

In NSCLC tissues, the expression of miR-30a-5p is considerably downregulated (128). Related studies showed that loss of CD73 significantly suppressed the flexibility of NSCLC cells in migrating, and CD73 repressed cell proliferation in NSCLC cells by moving speeding up cell cycle (125). CD73 has an important effect on tumor cells through the EGFR signaling pathway (35, 129). In NSCLC cells, miR-30a-5p can inhibit the expression of CD73 by directly binding to its 3′-UTR, and the ectopic expression of miR-30a-5p was significantly reduced while the expression of CD73 was increased (125). MiR-30a-5p targets the CD73 3′-UTR to inhibit its expression, which may be a good approach for NSCLC treatment.

Researchers have discovered that *KMT5A*, a downstream target of miR-340-5p, regulated CD8^+^ T cells and facilitated the immunosuppressive ability of DLBCL cells (130). The functional avidity of CD8^+^ T cells is associated with superior control of tumor growth (131). CD73-expressing tumor cells showed negative effects on the regulation of the antitumor T-cell response, which could also increase T-cell apoptosis (132). In LY-1 cells, silencing *KMT5A* enhanced the ubiquitination of CD73, which was reduced by COP1 knockdown, downregulating CD73 (130). The miR-340-5p/*KMT5A* axis plays an antitumor role on DLBCL cells independent of immune regulation (130). This provides a new perspective for the treatment of DLBCL.

### CircRNAs Target CD73

CircRNAs often act as miRNA sponges (133). CD73 has been demonstrated to promote tumor progression by suppressing the antitumor immune response, which is a new immune checkpoint related to adenosine metabolism (44). GAL-8, a member of the glycan-binding protein unit, was proposed to be able to induce the apoptosis of activated T cells and is involved with Th17 cells. It also promotes the differentiation of immunosuppressive Tregs, therefore showing immunosuppressive effects (134). CD73 and GAL-8 are targets of circHMGCS1-016 and miR-1236-3p in intrahepatic cholangiocarcinoma (ICC). A study showed that the proteins of CD73 and GAL-8 were highly expressed in ICC tissues and that they showed a negative correlation with CD8^+^ T cells (135). Furthermore, circHMGCS1-016 promoted ICC progression through miR-1236-3p/CD73 and GAL-8, acting as a sponge for miR-1236-3p (135). The regulation of CD73 and GAL-8 expressions by circHMGCS1-016 may be a novel therapy for ICC.

## The Impact of MicroRNA Targeting TIM-3 on Cancer

Human TIM-3 (T-cell immunoglobulin mucin receptor 3) is a specific supermolecule along with 301 amino acids and is encoded by *HAVCR2* (136).

Leukemic stem cells (LSCs) are abnormally proliferated in acute myeloid leukemia (AML), which is a complicated blood malignancy (137, 138). Both LSCs and biological process stem cells (hematopoietic stem cells, HSCs) are found in the bone marrow of AML patients. Among them, a characteristic of TIM-3 is its particular expression on LSCs (36, 37). TIM-3 may be considered as an acceptable candidate to eradicate AML LSCs (36). Based on flow cytometry analyses, miR-125a-3P and miR-133-5p showed a silencing phenomenon on the protein expression of TIM-3 in the HL-60 cell line (39, 139). The significant distinction of the silencing impact between the levels of informational RNAs and proteins of TIM-3 indicated that miR-125a-3p and miR-133a-5p may play restrictive roles in TIM-3 expression at the informational RNA change of location level by steric hindrance rather than informational RNA degradation to provide antitumor effects (39, 139).

## The Impact of MicroRNA Targeting ICOS on Cancer

Inducible co-stimulator (ICOS) and its ligand (ICOSL) operate as very important regulatory mechanism between immune and epithelium cells (140). ICOS is also an essential co-stimulatory molecule for T-cell function (141).

MiR-21 plays a key role in the regulation of the disease progression of B-cell lymphoma (38, 142). An experiment discovered a pre-B malignant lymphoid-like phenotype caused by the overexpression of miR-21 (143). MiR-21 induced the expression of ICOS on Tregs through the upregulation of p-STAT3, increased the interaction of Tregs with epithelial cells *via* the ICOS/ICOSL axis, stimulated neoplasm ontogenesis, and also led to the chemoresistance of B-cell lymphoma (144). Mir21 can also regulate the expressions of ICOS/ICOSL to affect the progression of CRC [36]. All these may provide novel treatment options for B-cell lymphoma.

ICOS is a marker of T-cell activation (145), and it was upregulated in the tumor tissues of obese patients with lung adenocarcinoma (LUAD) (141). Obesity mediates immune exhaustion through leptin (146). Studies have shown that miR-27a-3p could directly interact with ICOS and downregulate its expression in LUAD (141). IFN-γ is a significant activator of innate and adaptive immunity (147). However, the level of miR-27a-3p was obviously reduced in adipose-derived exosomes from obese individuals, which promoted ICOS^+^ T-cell proliferation, thereby influencing IFN-γ secretion and, hence, inhibiting the occurrence and development of LUAD (141). This provides new concept for the clinical treatment of LUAD.

## The Impact of NcRNAs Targeting CD276 on Cancer

Human B7-H3 (CD276) is a member of the B7/CD28 antibody taxonomic group that provides crucial co-stimulatory signals that regulate the functions of T-lymphocytes in growth regulation, infections, and immune response (148). 2Ig and 4Ig of human B7-H3 both repressed lymphocyte proliferation and protein production (149).

### MiRNAs Target CD276

CD276/B7-H3 has a high expression in solid tumors and leads to immune evasion and metastasis (40, 150). High expressions of CDK6 and CD276 have a negative influence on medulloblastoma survival, meaning that they play pro-oncogenic roles in medulloblastoma, supporting similar findings from prior studies (151). On the other hand, miR-1253 transfection plays a downregulated role in the expressions of CDK6 and CD276 (152). Silencing miR-1253 targets CDK6 and CD276 to suppress the growth of medulloblastoma (152). This provides good guidance to medulloblastoma therapy.

Previous studies indicated that CD276 appears to be involved in CRC progression and metastasis (41, 153). Moreover, CD276 is able to participate in miRNA-related regulation (154). CD276 is an immunoregulatory molecule that is directly targeted by miR-187 and is negatively related to miR-187 levels in CRC cells (45). The downregulation of miR-187 correlates with higher tumor grade and stage and functions as a tumor suppressor (155). Studies have shown that miR-187 was significantly downregulated in CRC tissues and its cell lines, and the overexpression of miR-187 can inhibit the proliferation, migration, and invasion of CRC cells and promote CRC cell apoptosis (45). The miR-187/CD276 axis provides a new potential therapeutic target for the treatment of CRC.

### CircRNAs Target CD276

Hsa_circ0021347 is a possible target regulated by B7-H3 in osteosarcoma (OS) cells, and its expression is reciprocally associated with the expression of B7-H3 (156). Analysis results have provided proof that B7-H3 accelerates growth cell immune escape through suppressing T-cell-mediated cellular immunity (157, 158). A previous study showed that miR-646 was involved in promoting the formation of OS (159). Bioinformatics analysis revealed that hsa_circ0021347 and miR-646 are involved in OS and that miR-646 potentially interacted with NOB1 (156). It can be inferred that the hsa_circ0021347–miR-646–NOB1 axis may be a good research direction for osteosarcoma treatment.

## Conclusion and Perspectives

The role of ncRNAs on immune checkpoints is complex. PD-1, PD-L1, CD47, BTLA, and the other immune checkpoints are regulated by miRNAs through directly combining with the 3′-UTR. CircRNAs can directly act on immune checkpoints, but most act as “miRNA sponge” to indirectly adjust them. LncRNAs and immune checkpoints interact to regulate the development of cancers. The three types of ncRNAs (miRNAs, circRNAs, and lncRNAs) have certain roles in promoting the study of tumor resistance and the development of new drug targets or immunotherapy options. Also, some of the specific roles of circRNAs and lncRNAs on immune checkpoints are not clearly elucidated and require further study. The targeted effect of ncRNAs on immune checkpoints is indicative of the development of immunotherapy for cancers.

## Author Contributions

YJ wrote the original draft and contributed to drawing. XY revised the manuscripts. XL provided ideas. LZ, YW, SD, and PC read and approved the final version of the manuscript. All authors contributed to the article and approved the submitted version.

## Funding

This work was funded by the Hengyang City Science and Technology Planning Project (grant no. 202150063473), the Scientific Research Project of Hunan Provincial Health Commission (grant no. 202202044140), the Scientific Research Project of Hunan Provincial Education Department (grant no. 21B0438), and the Hunan Province Cooperative Innovation Center for Molecular Target New Drug Study (grant no. 2014-405).

## Conflict of Interest

The authors declare that the research was conducted in the absence of any commercial or financial relationships that could be construed as a potential conflict of interest.

## Publisher’s Note

All claims expressed in this article are solely those of the authors and do not necessarily represent those of their affiliated organizations, or those of the publisher, the editors and the reviewers. Any product that may be evaluated in this article, or claim that may be made by its manufacturer, is not guaranteed or endorsed by the publisher.
